# Assessment of Fecal Microflora Changes in Pigs Supplemented with Herbal Residue and Prebiotic

**DOI:** 10.1371/journal.pone.0132961

**Published:** 2015-07-15

**Authors:** Ashis Kumar Samanta, C. Jayaram, N. Jayapal, N. Sondhi, A. P. Kolte, S. Senani, M. Sridhar, A. Dhali

**Affiliations:** 1 Feed Additives and Nutraceuticals Laboratory, National Institute of Animal Nutrition and Physiology, Adugodi, Hosur Road, Bangalore, 560030, India; 2 Omics Laboratory, National Institute of Animal Nutrition and Physiology, Adugodi, Hosur Road, Bangalore, 560030, India; 3 Fermentation Technology Laboratory, National Institute of Animal Nutrition and Physiology, Adugodi, Hosur Road, Bangalore, 560030, India; Graz University of Technology (TU Graz), AUSTRIA

## Abstract

Antibiotic usage in animals as a growth promoter is considered as public health issue due to its negative impact on consumer health and environment. The present study aimed to evaluate effectiveness of herbal residue (ginger, *Zingiber officinale*, dried rhizome powder) and prebiotic (inulin) as an alternative to antibiotics by comparing fecal microflora composition using terminal restriction fragment length polymorphism. The grower pigs were offered feed containing antibiotic (tetracycline), ginger and inulin separately and un-supplemented group served as control. The study revealed significant changes in the microbial abundance based on operational taxonomic units (OTUs) among the groups. Presumptive identification of organisms was established based on the fragment length of OTUs generated with three restriction enzymes (MspI, Sau3AI and BsuRI). The abundance of OTUs representing *Bacteroides intestinalis*, *Eubacterium oxidoreducens*, *Selonomonas sp*., *Methylobacterium sp*. and *Denitrobacter sp*. was found significantly greater in inulin supplemented pigs. Similarly, the abundance of OTUs representing *Bacteroides intestinalis*, *Selonomonas sp*., *and Phascolarcobacterium faecium* was found significantly greater in ginger supplemented pigs. In contrast, the abundance of OTUs representing pathogenic microorganisms *Atopostipes suicloacalis* and *Bartonella quintana str*. *Toulouse* was significantly reduced in ginger and inulin supplemented pigs. The OTUs were found to be clustered under two major phylotypes; ginger-inulin and control-tetracycline. Additionally, the abundance of OTUs was similar in ginger and inulin supplemented pigs. The results suggest the potential of ginger and prebioticsto replace antibiotics in the diet of grower pig.

## Introduction

The principal aim of livestock production is the delivery of safe and healthy food for human consumption taking into account the welfare of animals, consumer awareness, public health, environmental issues etc. Following the discovery of growth promoting effect of antibiotics in 1940s, its application as an animal feed supplement became highly popular worldwide and continued subsequently for several decades [1]. However, the extensive use of feed antibiotics over a period of time strongly contributed to the emergence of many drug resistant microbes. In view of the above facts, the World Health Organization [2] and the Economic and Social Committee of the European Union [3] declared the inclusion of antimicrobials in animal feed as a public health issue. Consequently, the European Union banned the use of antibiotics as growth promoters in food animal since 2006 [1].

The restriction on the use of antibiotics as growth promoters in feed has put severe limitations on swine industry; one of them is substantial increase in the use of antibiotics for therapeutic purpose [4]. This necessitated worldwide research for identifying viable alternatives to replace antibiotics after considering animal welfare and consumer issues [5]. In this context, herbal residue and prebiotic can be possible alternatives. The benefits of herbals and their extracts is well recognized and have been related to the modulation of gut microbiota in addition to improvement of digestibility, stimulation of the immune system, antimicrobial activities and, anti-inflammatory and antioxidant properties [6]. Ginger is a commonly used herb and widely popular for its medicinal values. Ginger has been found effective in killing the gut pathogen *Helicobacter pylori* [7]. In a recent report, the effectiveness of Zingiberis residue in inhibiting pathogens and its possible use as an alternate to antibiotics in pigs has been indicated [8]. Similarly, inulin feeding has been directly related to gut microbial composition and performance in pigs [9–16].

The gastrointestinal tract of animal harbors a vast population of microflora that exceeds the total number of host cells [17]. Yet, the microflora composition is poorly understood due to our inability to culture most members of the gut microflora community that has led to earlier studies considering only a fraction of gastrointestinal microbes [18]. Among the several culture independent methods, terminal restriction fragment length polymorphism (T-RFLP) analysis is considered as an effective and high throughput fingerprinting technique to monitor the changes in the structure and composition of microbial communities [19].

Despite historical evidences for extensive application of plant sourced bioactive compounds by ancient civilization of India and China [20], these were kept aside due to revolutionary applications of numerous antibiotics and chemotherapeutic agents to manage and treat diseases in both human and animals during previous century. Phobia against antibiotics and chemotherapeutic agents in the present time garnered greater research interest for searching the effective alternatives. In this direction, herbal residues and prebiotics are emerging as front runner to occupy the vacant space of antibiotics as feed additives for food animals during the post-antibiotic-ban-era around the world. Nevertheless, our understanding on the response of gut microflora in pigs to either herbal residue or prebiotic is incomplete and inadequate. Therefore, the present study aimed to assess the shift in hindgut microflora composition in grower pigs in response to the supplementation of antibiotic (tetracycline), herbal residue (ginger, *Zingiber officinale*) and prebiotic (inulin). The compositional changes in microflora communities were assessed by T-RFLP analysis as this technique is highly reproducible and enables a “snap shot” view of the complex bacterial population of the gut at any particular time [21, 22].

## Materials and Methods

### Animals and diet

The experiment was conducted at the facility of All India Coordinated Research Project on Pigs, College of Veterinary Sciences, Sri Venkateswara Veterinary University, Tirupati, India. Sixteen crossbred pigs (Large White Yorkshire×Non-descript) with an average body weight of 15.8±1.1 kg were divided into four groups with four animals in each. The animals were maintained for three months on basal diet comprising of maize 54 parts, soybean meal 22 parts, de-oiled rice bran 21 parts, mineral mixture 2 parts and salt 1 part. The animals had free access to feed and water during the experimental period. The animals of control group were offered the basal feed without any additives. In contrast, the basal diet was supplemented with 0.25% tetracycline, 2% ginger residue (powder of ginger rhizome after juice extraction, procured from M/S Chemiloids Herbs, Pvt. Ltd., Vijayawada, India) or 2% inulin (procured from M/S Deena Enterprises, Hyderabad, India) in the experimental groups. Fresh fecal samples were collected from each animal after three months of feeding to study the microbial community composition in each group. Fecal samples were stored at -20°C until further analysis. All the experimental protocols and animal care met the regulations and was approved by the “Institute Animal Ethics Committee, College of Veterinary Sciences, Sri Venkateswara Veterinary University, Tirupati, India”.

### DNA extraction

Total DNA was extracted from the fecal samples (~200 mg) collected from individual pigs using ZR fecal DNA extraction kit (Zymo research, USA) as per the manufacturer’s recommendations.

### T-RFLP analysis

The purified DNA was used to amplify the microbial 16S rRNA gene sequences with universal primers 27F [23] and 907R [24]. The forward primer was 5’ labeled with 6-carboxyfluorescein (FAM). PCR amplifications were carried out in a total volume of 20 μl containing 10 μl 2× PCR master mix with MgCl_2_ (Cat. No. P4600, Sigma Aldrich, USA), 1 μl (10 μM) of 5^’^ labeled 27F and 907R, 100 ng of DNA and nuclease free water. The PCR reactions were run in duplicate in an Eppendorf Mastercycler with the following amplification conditions: initial denaturation at 94°C for 5 min followed by 40 cycles of denaturation at 94°C for 45s, annealing at 57°C for 45s, and extension at 72°C for 1 min, with a final extension step at 72°C for 5 min. The specificity of PCR products was analyzed by gel electrophoresis on a 1% agarose gel and visualized after staining with ethidium bromide. Approximately 6μl of PCR products were digested with the restriction enzymes MspI, Sau3AI, and BsuRI in duplicates (Fermentas, USA) to generate labeled terminal restriction fragments (T-RFs). Restriction digestion was performed in a total volume of 15 μl containing 6 μl of PCR product, recommended buffer, specific enzyme and nuclease free water, and incubated at 37°C for 4 h, followed by enzyme inactivation at 65°C for 15 min. Representative samples were checked for digestion on 1% agarose gel. The T-RFs generated were separated by capillary electrophoresis on ABI 3730 automated DNA analyzer. The lengths of the fluorescently labeled T-RFs were recorded after comparing with an internal standard and data were analyzed using Gene Mapper Software (Applied Biosystems, USA). T-RFs were defined as peaks with a size of x ±2bp within the pseudo replicates of samples and rounded to the nearest even number between samples to produce OTUs (S1 Data).

### Identification of OTUs

Microbial identification of each OTU was done at Microbial Community Analysis III (MiCA3) Virtual Digest (http://mica.ibest.uidaho.edu/digest.php) [25]. The *in-silico* database was constructed online with Eub-8F and Eub-926R primer pairs, restriction enzymes MspI, Sau3AI, and BsuRI using “Pig Bacterial 16S rRNA”, “H.Q. 16S Gut Organisms” and “Mammalian Gut Microbes 16s rRNA” datasets available at MiCA3 portal.

### Statistical analysis

The raw T-RFLP data obtained from all the experimental groups were initially pretreated as follows, to make the data compatible for statistical analysis. The OTUs with <30 bp length were discarded. Phylotype with peak height <50 and relative peak area <1% were excluded. The fragments differing by <2 bp were considered as single phylotype and their abundance values were merged for the analysis. Percentage of abundance of OTUs was calculated for each fragment based on peak area value.

The abundance data was statistically analyzed by R-Statistical package (3.0.2). Multivariate data analyses were carried out using PRIMER6, PRIMER-E ltd., UK [26]. These analyses were used to examine the OTU abundance within each treatment group and relative abundance among the different treatment groups. Bray-Curtis measure of similarity [27] was calculated to examine similarities among fecal bacterial communities of the experimental groups from the OTU data matrices. One-way analysis of similarity (ANOSIM; [28]) was used to test if fecal bacterial communities were significantly different among the groups. The R- value describes the extent of similarity between the different pairs of treatments in the ANOSIM analysis. R-value close to the unity indicates that two groups are different and a value close to zero indicates similarity between the groups.

Similarity percentage (SIMPER; [28]) analyses were performed to determine the overall average similarity in fecal bacterial community composition of the experimental groups, as well as to determine which OTU contributed greatest to the dissimilarity. The overall average dissimilarity between bacterial communities was calculated and the average contribution of the i^th^ OTU to the overall dissimilarity was determined. Average abundance of the OTUs in each group was determined and the OTUs contributing significantly to the dissimilarity between the groups were identified. The percent contributions of individual OTU and the cumulative percent contribution to the top 50% of the average dissimilarities were also calculated. Unconstrained ordinations using non-metric multidimensional scaling (nMDS) were done to illustrate the relationships among the groups [29, 30]. The nMDS ordinations show the relationships among the groups using the ranks of similarities.

## Results

**Fig 1:**
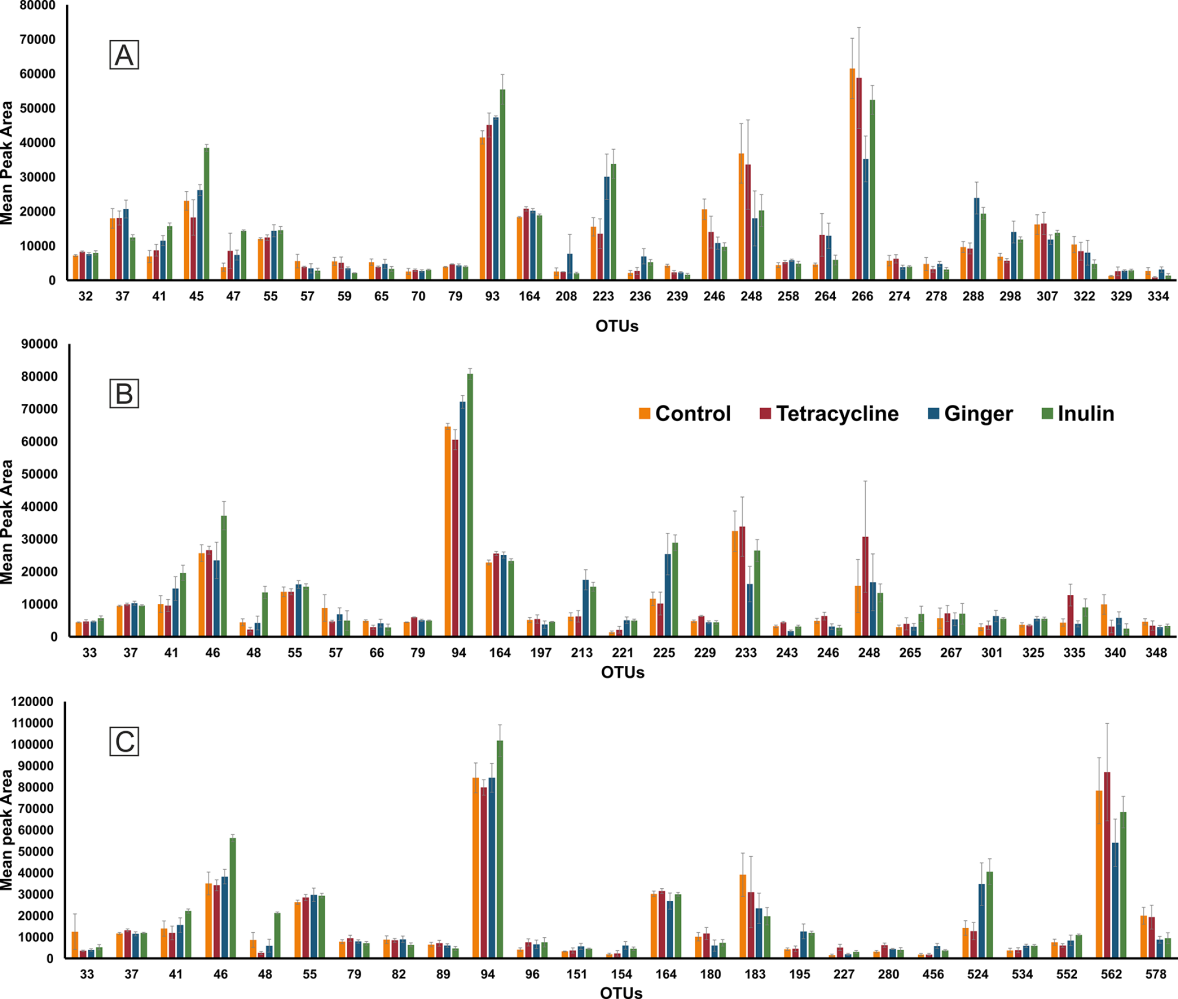
Histogram showing abundance response of 16s rRNA OTUs generated by MspI (A), Sau3AI (B) and BsuRI (C) restriction enzymes. The 16s rRNA gene was amplified from fecal microflora of control or tetracycline, ginger and inulin supplemented pigs.

In the present study, the experimental animals were either fed basal diet or basal diet supplemented with tetracycline, ginger or inulin for three months. At the end of the feeding period, fecal samples were collected from all the experimental groups to monitor the hindgut bacterial fingerprint by T-RFLP. The T-RFLP profiles revealed varied number of OTUs; 30 OTUs with MspI, 27 OTUs with Sau3AI and 25 OTUs with BsuRI restriction enzymes. The length of OTUs was ranged from 32–334 bp, 33–348 bp and 33–578 bp for MspI (Fig 1A), Sau3AI (Fig 1B) and BsuRI (Fig 1C), respectively. The OTUs with similar fragment length from all the restriction enzymes were found to be 41, 55, 79 and 164 bp.

### Effect of supplementation on OTUs abundance

Two OTUs (93 and 266 bp) generated with MspI and one OTU (94 bp) generated with Sau3AI exhibited maximum abundance across the treatment groups.

A significant variation in the abundance of OTUs generated with the three restriction enzymes was observed in inulin supplemented as compared to control group. Eight, six and seven OTUs generated respectively with MspI, Sau3AI and BsuRI exhibited (p<0.05) greater abundance, and one OTU generated with either MspI or BsuRI exhibited (p<0.05) lesser abundance in response to inulin supplementation as compared to control. The comparison between the inulin and tetracycline supplementations revealed a significantly (p<0.05) greater abundance of three, six and eight OTUs generated respectively with MspI, Sau3AI, and BsuRI in response to inulin as compared to tetracycline supplementation. In contrast, one and three OTUs generated respectively with MspI and Sau3AI exhibited significantly (p<0.05) lesser abundance as a result of inulin as compared to tetracycline supplementation. Similarly, three OTUs generated with MspI and two OTUs generated with Sau3AI or BsuRI exhibited significantly (p<0.05) greater abundance in inulin as compared to ginger supplementation. In contrast, two OTUs generated with MspI exhibited significantly (p<0.05) lesser abundance in inulin as compared to ginger supplemented group.

A significant variation in the abundance of OTUs generated with the three restriction enzymes was observed in response to ginger supplementation as compared to control. It was observed that three OTUs generated with either MspI or Sau3AI exhibited significantly (p<0.05) greater abundance in ginger supplemented as compared to control group. In contrast, the abundance of three and one OTUs generated respectively with MspI and Sau3AI was found significantly (p<0.05) lesser as a result of ginger supplementation as compared to control. Similarly, the supplementation of ginger was found to influence the abundance of OTUs significantly as compared to tetracycline supplementation. Evidently, three OTUs generated with MspI or Sau3AI and one OTU generated with BsuRI exhibited (p<0.05) greater abundance in response to ginger as compared to tetracycline supplementation. Conversely, four OTUs generated with Sau3AI exhibited (p<0.05) lesser abundance in ginger as compared to tetracycline supplemented group.

The supplementation of tetracycline significantly (p<0.05) increased the abundance of three OTUs generated with MspI or Sau3AI and two OTUs generated with BsuRI as compared to control. Further, one OTU generated with MspI or Sau3AI exhibited significantly (p<0.05) lesser abundance in response to tetracycline supplementation as compared to control.

### Comparativeresponse among the treatment groups

**Fig 2:**
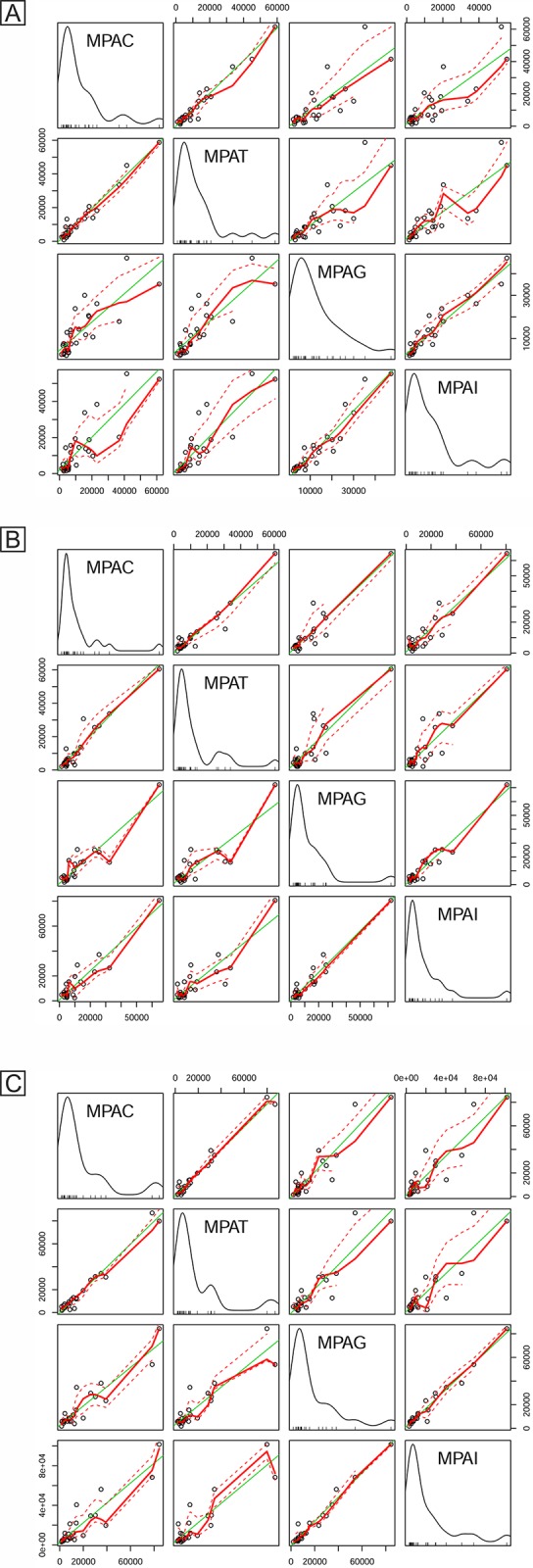
Scatter plot matrix showing the relative response of the 16s rRNA gene OTUs generated by MspI (A), Sau3AI (B) and BsuRI (C) restriction enzymes from pig fecal samples. MPAC-Mean peak area of control pigs, MPAT—Mean peak area of tetracycline supplemented pigs, MPAG- Mean peak area of ginger supplemented pigs, MPAI- Mean peak area of inulin supplemented pigs.

Albeit significant difference in the abundance response was observed among the treatment groups for the three restriction enzymes MspI, Sau3AI, and BsuRI, scatter plot matrix of the abundance data (mean peak area of OTUs) revealed a positive linear relationship for control *vs*. tetracycline and ginger *vs*. inulin. It also revealed a weak positive linear relationship between control *vs*. ginger, control *vs*. inulin, tetracycline *vs*. ginger and tetracycline *vs*. inulin (Fig 2).

### Microbial community analysis

**Fig 3:**
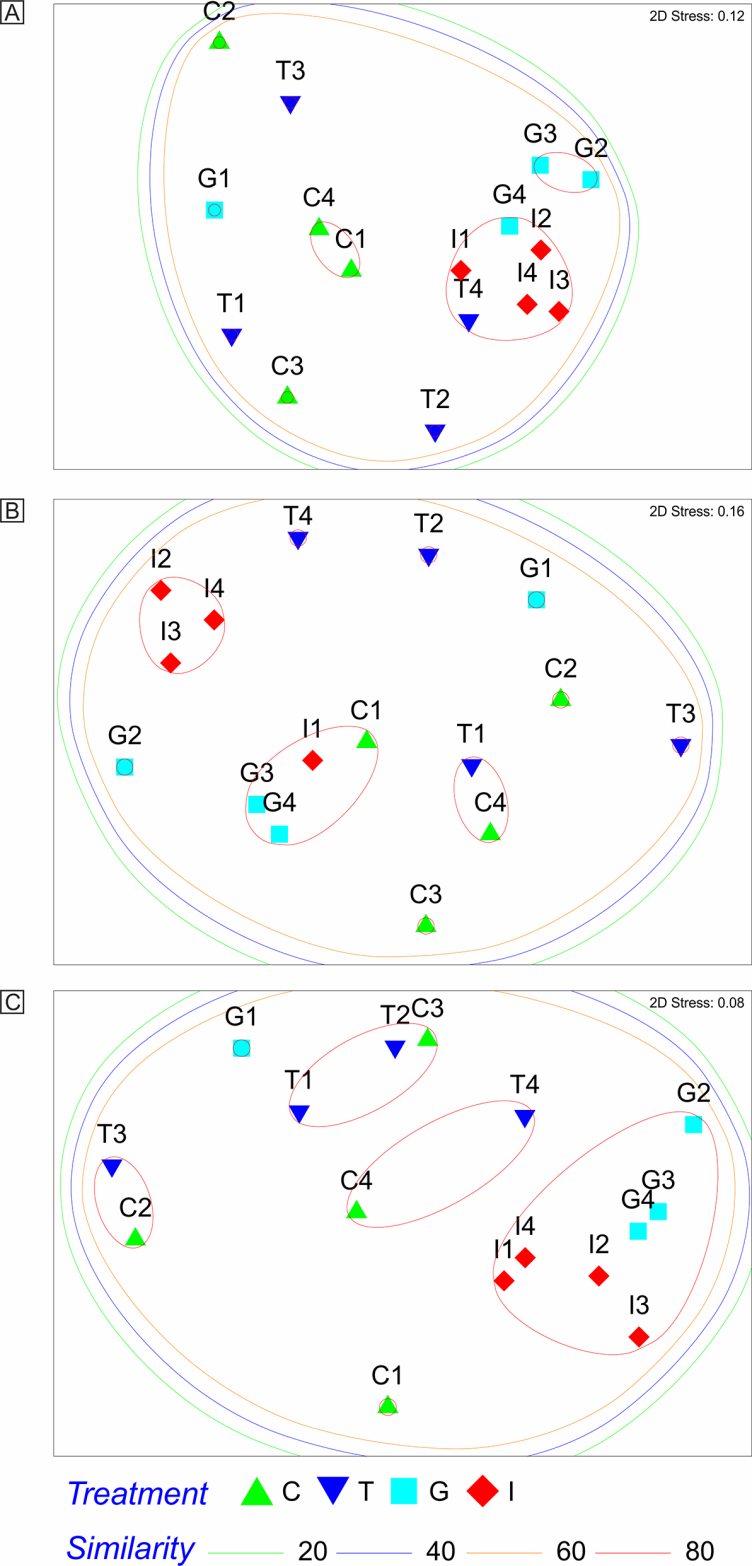
Non-metric multi dimensional analysis of OTU abundance showing relationship among the individuals of four treatment groups. The OTUs were generated from 16s rRNA gene amplified from pig fecal microflora of control or tetracycline, ginger and inulin supplemented groups. A: OTUs generated with MspI, B: OTUs generated with Sau3AI, C: OTUs generated BsuRI.

A shift in the microbial community composition was noticed with ginger and inulin supplementations and the results indicated clustering of two major phylotypes (i)ginger-inulin and (ii)control-tetracycline (Fig 3, S1 Fig).

The results of the Analysis of Similarity (ANOSIM) are presented in Table 1. Although no significant dissimilarity was observed among the OTUs considering all treatments, specific treatment combinations were found to be significantly (p<0.05) dissimilar in all the restriction enzymes. The phylotypes of control *vs*. inulin combination generated with all the restriction enzymes exhibited significant (p<0.05) dissimilarity. Evidently, phylotypes of treatment combination tetracycline *vs*. inulin generated with Sau3AI, and BsuRI exhibited significant (p<0.05) dissimilarity, while phylotypes of the same combination generated with MspI exhibited significant (p<0.05) similarity. The phylotypes of ginger *vs*. inulin combination generated with MspI and BsuRI exhibited significant (p<0.05) similarity.

**Table 1:** Analysis of Similarity (ANOSIM) among OTUs generated from 16s rRNA gene amplified from pig fecal microflora among treatment combinations.

		Global		Group Wise		
Enzyme	Treatment Combination	R Value	P Value	R Value	P Value	Inference
MspI	All treatments	0.338	0.002			++
	Control and inulin			0.729	0.029	+
	Tetracycline and inulin			0.438	0.029	++
	Ginger and inulin			0.396	0.029	+++
	Ginger and tetracycline			0.396	0.057	++
	Ginger and control			0.479	0.086	++
	Tetracycline and control			0.010	0.514	++++
Sau3AI	All treatments	0.424	0.001			++
	Control and inulin			0.646	0.029	+
	Tetracycline and inulin			0.688	0.029	+
	Ginger and inulin			0.198	0.171	+++
	Ginger and tetracycline			0.552	0.057	+
	Ginger and control			0.281	0.086	++
	Tetracycline and control			0.240	0.086	++
BsuRI	All treatments	0.336	0.001			++
	Control and inulin			0.521	0.029	+++
	Tetracycline and inulin			0.677	0.029	+
	Ginger and inulin			0.375	0.029	++
	Ginger and tetracycline			0.250	0.114	++
	Ginger and control			0.250	0.143	++
	Tetracycline and control			0.021	0.429	++++

+ Less similar, ++ Similar, +++ More similar and ++++ Most Similar.

Similarity percentage among the treatment groups and phylotypes of individual treatment groups were calculated using SIMPER for the three restriction enzymes. Phylotypes of inulin supplemented pigs exhibited highest similarity as compared to the supplementation of ginger or tetracycline for all the restriction enzymes used. Irrespective of the restriction enzymes, the average similarity percentage of OTUs for all the groups is presented in Table 2.

**Table 2:** Similarity Percentage (SIMPER) showing average similarity exhibited by the phylotypes of four treatment groups generated with three restriction enzymes MspI, Sau3AI and BsuRI.

Restriction enzyme	Treatment group	Average similarity (%)
MspI	Control	76.09
	Tetracycline	72.98
	Ginger	76.40
	Inulin	86.34
Sau3AI	Control	77.08
	Tetracycline	74.11
	Ginger	74.75
	Inulin	81.10
BsuRI	Control	73.97
	Tetracycline	75.23
	Ginger	75.23
	Inulin	85.81

The average dissimilarity of OTUs between the treatment combinations is presented in the Table 3. In case of the restriction enzyme MspI, the dissimilarity (%) varied from 22.76 (ginger *vs*. inulin) to 28.83 (control *vs*. inulin). Similarly the dissimilarity (%) varied from 23.77 (ginger *vs*. inulin) to 31.08 (control *vs*. inulin) for Sau3AI and 22.48 (ginger *vs*. inulin) to 29.49 (control *vs*. ginger) for BsuRI.

**Table 3:** Average dissimilarity among the OTUs across the treatment groups.

Enzyme	Treatment combinations	Average dissimilarity (%)
MspI	Control *vs*. tetracycline	26.37
	Control *vs*. ginger	29.00
	Control *vs*. inulin	28.83
	Tetracycline *vs*. ginger	28.74
	Tetracycline *vs*. inulin	25.70
	Ginger *vs*. inulin	22.76
Sau3AI	Control *vs*. tetracycline	25.74
	Control *vs*. ginger	26.94
	Control *vs*. inulin	31.08
	Tetracycline *vs*. ginger	29.19
	Tetracycline *vs*. inulin	30.04
	Ginger *vs*. inulin	23.77
BsuRI	Control *vs*. tetracycline	25.22
	Control *vs*. ginger	29.49
	Control *vs*. inulin	29.22
	Tetracycline *vs*. ginger	27.64
	Tetracycline *vs*. inulin	29.35
	Ginger *vs*. inulin	22.48

### Putative identification of OTUs according to MiCA3 Virtual Digest

The microorganisms identified from the MiCA3 virtual digest are presented in Table 4. Most of the OTUs generated with the three restriction enzymes could not be assigned to any known microorganism. Nevertheless, seven OTUs generated with MspI were identified as beneficial microbes and the corresponding number was one for Sau3AI and three for BsuRI. In contrast, three and one OTUs generated respectively with Sau3AI and BsuRI were identified as pathogens.

**Table 4:** Putative identification of OTUs according to MiCA3 virtual digest (http://mica.ibest.uidaho.edu/digest.php) and abundance of the identified organisms. C: Control, T: tetracycline supplementation, G: ginger supplementation, I: inulin supplementation.

OTU	Treatment combinations						Enzyme	Organism identified [Reference for categorization]
	C *vs*. T	C *vs*. G	C *vs*. I	T *vs*. G	T *vs*. I	G *vs*. I		
Beneficial organisms								
32	↑*	↑	↑	↓	↓	↑	MspI	*Butyrivibrio fibrisolvens* [31]
79	↑*	≈	≈	↓	↓*	≈	MspI	*Parabacteroides distasonis* [32]
93	↑	↑*	↑*	↑	↑	↑	MspI	*Bacteroides intestinalis* [33]
151	≈	↑	↑*	↑	↑	↓	BsuRI	*Denitrobacter sp*. [34]
164	↑*	↑*	≈	↓	↓	↓	MspI	*Phascolarctobacterium faecium* [35]
195	≈	↑	↑*	↑	↑*	↓	BsuRI	*Methylobacterium sp*. [36]
223	↓	↑	↑*	↑	↑*	↑	MspI	*Eubacterium oxidoreducens* [37]
280	↑*	↑	↑	↓	↓	↓	BsuRI	*Lactobacillus ruminis* [38]
288	↓	↑*	↑*	↑	↑*	↓	MspI	*Selenomonas sp*. [39]
298	↓	↑	↑*	↑*	↑*	↓	MspI	*Selenomonas infelix* [40]
325	≈	↑	↑	↑	↑	≈	Sau3AI	*Anaerovibrio lipolyticus* [41]
Pathogenic organisms								
66	↓*	↓	↓	↑	≈	↓	Sau3AI	*Flavobacterium sp*. [42]
227	↑*	≈	↑	↓	↓	↑	BsuRI	*Paenibacillus sp*. [43]
229	↑*	↓	↓	↓*	↓*	≈	Sau3AI	*Atopostipes suicloacalis* [44]
243	↑	↓*	≈	↓*	↓	↑	Sau3AI	*Bartonellaquintana str*. *Toulouse* [45]

↑ Increased, ↓ Decreased, ≈ No change

* significant change (p<0.05)

It was observed that the abundance of four beneficial microbes (OTUs 93, 223, 298 and 288) was significantly (p<0.05) increased in response to inulin or ginger supplementation as compared to control or tetracycline. Similarly, the abundance of two beneficial microbes (OTUs 151 and 195) was significantly (p<0.05) increased in response to inulin as compared to control or tetracycline. Evidently, the abundance of two beneficial microbes (OTUs 32 and 280) was significantly (p<0.05) increased in response to tetracycline as compared to control. On the other hand, the abundance of the beneficial microbe corresponding to the OTU 79 was significantly (p<0.05) increased in response to tetracycline as compared to control and decreased in response to inulin as compared to tetracycline. While, the abundance of the beneficial microbe corresponding to the OTU 164 was significantly (p<0.05) increased in response to tetracycline and ginger as compared to control. The abundance of the microbe corresponding to the OTU 325 was not different in any treatment combinations.

The abundance of the pathogens represented by the OTU 243 was significantly (p<0.05) decreased in response to ginger as compared to control and tetracycline. Similarly, a significant (p<0.05) reduction in the abundance of the pathogen represented by the OTU 229 was observed in response to ginger and inulin as compared to tetracycline, but its abundance was significantly (p<0.05) increased with tetracycline supplementation as compared to control. Evidently, the pathogens represented by the OTUs 66 and 227 respectively decreased and increased significantly (p<0.05) in response to tetracycline as compared to control.

## Discussion

The study aimed to assess the efficacy of herbal residue (ginger) and prebiotic (inulin) as an alternative to replace antibiotic in the feed of grower pigs for maintaining favourable gut microflora composition. Ginger, inulinand tetracycline were supplemented in the diet of grower pigs and the microbial composition was assessed in the fecal samples through T-RFLP analysis. The results indicate that inulin and ginger can be potential alternatives in replacing antibiotic as feed supplement.

T-RFLP is a powerful alternative molecular tool for assessing the diversity of bacterial community structure from different ecosystems. It provides sensitive and rapid means to assess community diversity and obtain distinctive fingerprint of a microbial community [46]. In contrast, the potential disadvantage of the technique is OTU homoplasy that results in masking of community members those share the OTU of same length leading to underestimation of the community diversity [47]. Nevertheless, previously, the technique has been used successfully to establish the microbial diversity in mouse intestine [48], human feces [49], chicken gut [21] and pig gut [50]. In the current study, using the T-RFLP technique, we demonstrated a significant shift in the gut microbial composition in grower pigs in response to inulin, ginger and tetracycline supplementations as compared to control. A diverse number of OTUs were generated using the restriction enzymes MspI, Sau3AI and BsuRI, and the abundance of OTUs was varied significantly among the treatments. Several previous reports indicate that the use of more than one restriction enzyme provides better resolution of bacterial diversity [46, 51]. It has been shown earlier that MspI is suitable for T-RFLP application as it most often resolves individual populations [52]. In the current study, two additional enzymes Sau3AI and BsuRI were used. It was observed that although maximum numbers of OTUs (30) were generated with MspI followed by Sau3AI (27) and BsuRI (25), the numbers were comparable among the enzymes. The results indicated that the efficacy of all the three enzymes was nearly similar in assessing the microbial diversity in pig fecal samples.

Over the last two decades, a widespread interest has been developed among the researchers for developing feeding strategies to improve the gut health in food animals while minimizing in-feed antibiotic usage. The driving factor for such effort is the emerging evidence that antibiotic administration has a negative impact on host microbiota, immunity and health, and may spread the antibiotic resistance gene to pathogens [53]. Prebiotic has the ability to modulate gut health through an array of interactions with the intestinal epithelium, mucus layer, immune system and microbiota [14]. Further, it has been indicated that the ginger residue is effective in inhibiting the growth of pathogens and can be used as an alternate to dietary antibiotic supplementation in pigs [8]. Therefore, in the current study, the efficacy of inulin and ginger was assessed as an alternative to dietary antibiotic supplementation in grower pigs. It was observed that the composition of the gut microbial community could be altered significantly in grower pigs with the inclusion of inulin and ginger in the diet. Interestingly, the clustering analysis demonstrated the formation of two prominent phylotypes, ginger-inulin and control-tetracycline. The results indicated that the inulin and ginger supplementations yielded similar gut microbial composition, but these were different than that noticed in control and tetracycline supplemented groups. In addition, the ANOSIM analysis of the abundance of OTUs between the treatment combinations also revealed a significant effect of inulin and ginger supplementations on gut microbial composition.

Efforts were made to identify the organisms from the generated OTUs using MiCA3 virtual digest database. Eleven beneficial (*Butyrivibrio fibrisolvens*, *Parabacteroides distasonis*, *Bacteroides intestinalis*, *Denitrobacter sp*., *Phascolarctobacterium faecium*, *Methylobacterium sp*., *Eubacterium oxidoreducens*, *Lactobacillus ruminis*, *Selenomonas sp*., *Selenomonas infelix* and *Anaerovibrio lipolyticus*) and four pathogenic microorganisms (*Flavobacterium sp*., *Paenibacillus sp*., *Atopostipes suicloacalis* and *Bartonella quintana str*. *Toulouse*) were identified. The results indicated a significant increase in the abundance of the beneficial microbes *Bacteroides intestinalis*, *Selenomonas infelix*, *Selenomonas sp*. and *Eubacterium oxidoreducens* with inulin or ginger supplementation as compared to control or tetracycline supplementation. Additionally, inulin supplementation alone significantly increased the abundance of the beneficial microbes *Methylobacterium sp*. and *Eubacterium oxidoreducens* as compared to control or tetracycline supplementation. Interestingly, a significant reduction in the abundance of the pathogenic microorganisms was evident with the supplementation of inulin or ginger as compared to control or tetracycline.

In pigs, dietary supplementation of inulin is widely used to modify the gut microbiota composition for better performance [9, 10]. All types of inulin promotes a favourable microbial balance in the intestinal tract of pigs with increase in beneficial Bifidobacteria and Lactobacillus and concomitant decrease in less desirable populations such as Clostridia and members of Enterobacteriaceae [11]. It has been demonstrated that inulin-type fructans support higher bacterial diversity and a more rapid stabilization of the bacterial community in the gut of piglets [12]. Inulin also helps in stabilizing the gut microflora in case of bowel disorder. The supplementation of oligofructose in the diet of piglets suffering from induced diarrhoea with cholera enterotoxin has revealed an increase in lactobacilli abundance with simultaneous reduction in the population of harmful Enterobacteria in both ceacum and colon [13]. Antimicrobial properties of medicinal and aromatic herbs and their extracts are well known and documented. In pigs, the antimicrobial potential of herbal extracts to replace antibiotics in feeds is proven [54, 55]. It is reported that the ginger residue is effective in inhibiting the growth of pathogens and it can reduce the pathogenic microbial load in the large intestines of finisher pigs [8]. In current study, many beneficial microorganisms exhibited significantly increased response to ginger and inulin supplementations in pigs. In contrast, the abundance of the pathogenic microorganisms was decreased significantly in response to ginger or inulin supplementation. Moreover, a distinct phylotype arising from the ginger or inulin supplementation was evident that indicated their similar efficiency in promoting the favourable gut microflora and inhibiting pathogenic microorganisms.

In conclusion, the overall findings of the study indicated that inulin and ginger supplementations in the diet of grower pigs were effective in establishing a favourable gut microbial composition. Therefore, our findings agree with the hypothesis that herbal residue or prebiotic can be used as an effective alternative to replace antibiotic in the feed of grower pigs.

## Supporting Information

S1 Data Table containing OTUs generated with different restriction enzymes for individual samples. (XLSX)

S1 Fig Cluster analysis of OTU abundance data generated with different restriction enzymes. (TIF)
