# Supplementary figures and images for: Assessment of Fecal Microflora Changes in Pigs Supplemented with Herbal Residue and Prebiotic
Source: PLoS One. 2015 Jul 15;10(7):e0132961. doi: 10.1371/journal.pone.0132961 (PMC4503616; doi:10.1371/journal.pone.0132961)

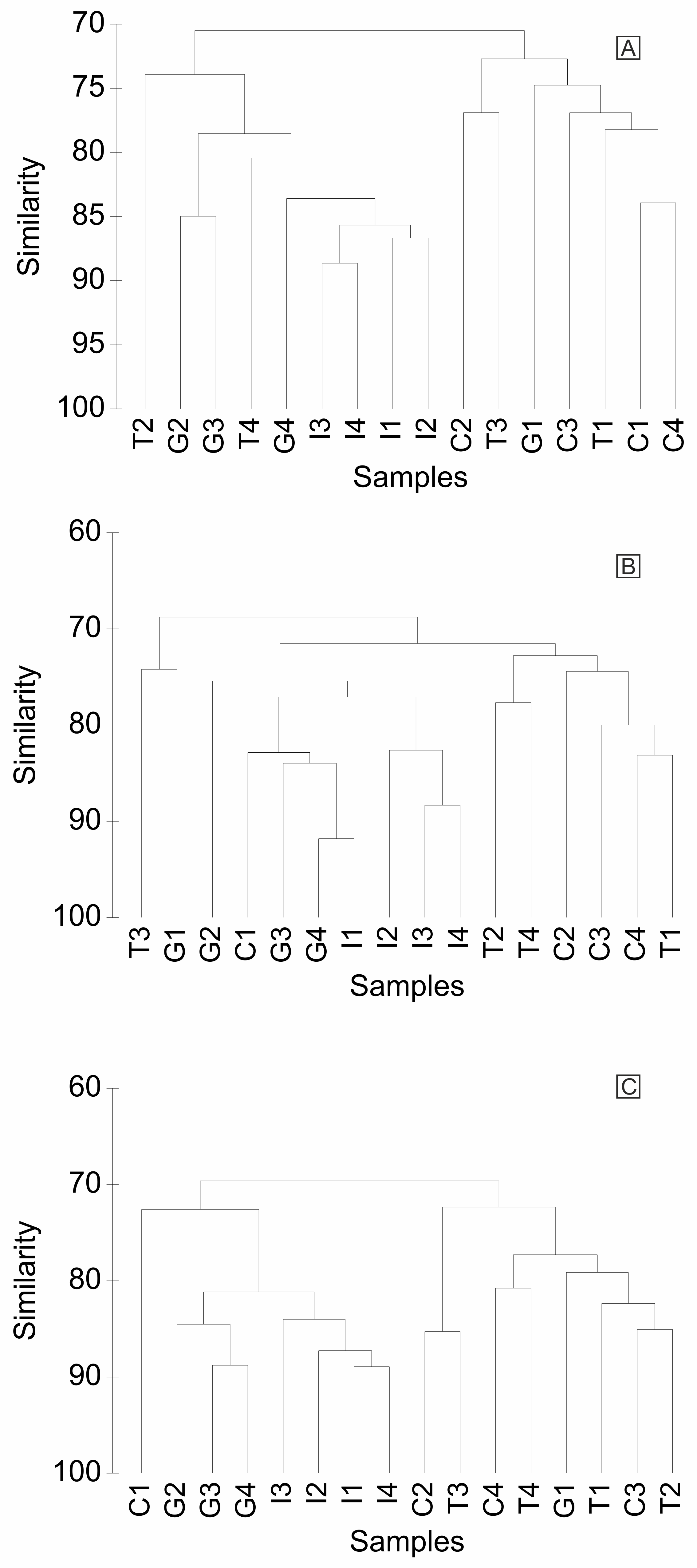

Supplement: S1 Fig — (TIF) [file pone.0132961.s002.tif]
